# Rapid determination of heterocyclic amines in ruminant meats using accelerated solvent extraction and ultra-high performance liquid chromatograph–mass spectrometry

**DOI:** 10.1016/j.mex.2019.11.014

**Published:** 2019-11-18

**Authors:** Charles F. Manful, Natalia P. Vidal, Thu H. Pham, Muhammad Nadeem, Evan Wheeler, Melissa C. Hamilton, Karen M. Doody, Raymond H. Thomas

**Affiliations:** School of Science and the Environment/Boreal Ecosystem Research Initiative, Grenfell Campus, Memorial University of Newfoundland, Corner Brook, A2H 5G4, Canada

**Keywords:** Heterocyclic amines determination by ASE–UHPLC-HRAMS, Heterocyclic amines, Carcinogens, Chromatography, Mass spectrometry, Pressurized solvent extraction, Grilled meat safety, Moose meat

## Abstract

Cooking techniques such as grilling confer several benefits to meat during food preparation including improved palatability, digestibility, preservation, and safety, as well as enhancing the sensory characteristics and net nutritional gain. However, grilling can lead to the formation of harmful compounds such heterocyclic amines (HCAs). HCAs are potent carcinogenic and mutagenic nitrogen containing compounds produced during certain cooking conditions of protein rich foods. Dietary intake of HCAs is associated with increased risk factors for cancers in humans. As such, there is overwhelming interest in identifying improved methods for rapid and accurate determination of heterocyclic amines in food matrices that is sensitive and avoids exhaustive sample preparation steps. Herein, we describe an approach that involves first extracting HCAs by pressurized accelerated solvent extractor using methanol as solvent, followed by addition of internal standard and quantification of HCAs by ultra-high performance liquid chromatography-high resolution accurate mass spectrometric detection (UHPLC-HRAMS). This method is fast, accurate, reproducible and does not require exhaustive sample pre-treatments prior to UHPLC-HRAMS analysis compared to existing/traditional methods for HCA analysis.

• The method is automated, fast and uses tunable pressurized liquid extractor to selectively extract HCAs
• Method does not require exhaustive cleanup and preconcentration steps prior to UHPLC/HRAMS analysis of HCAs
• Validation showed method to be accurate, precise, and useful for routine multi-sample HCA analyses

## Graphical abstract

**Figure:**
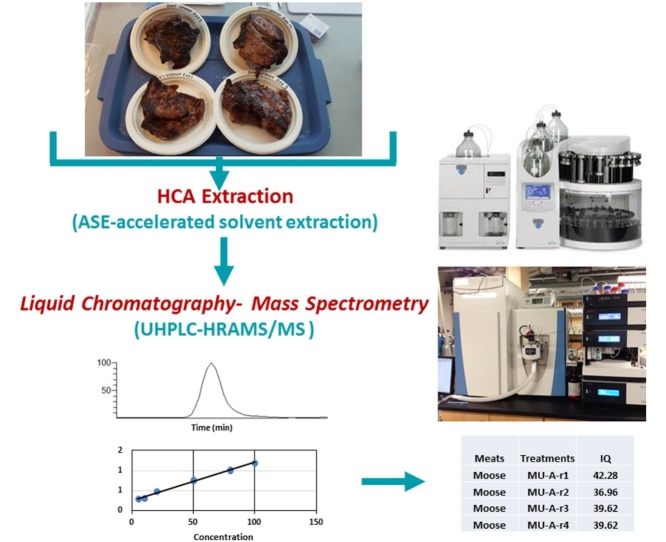

**Specification Table**

**Table:** 

Subject Area:	Chemistry
More specific subject area:	Food Science Food Safety and Quality
Method name:	Heterocyclic amines determination by ASE–UHPLC-HRAMS
Name and reference of original method:	[1] Ouyang, Yun-fu, Li, Hai-bo, Tang, Hong-bing, Jin, Yi and Li, Gui-ying, A reliable and sensitive LCMS-IT-TOF method coupled with accelerated solvent extraction for the identification and quantitation of six typical heterocyclic aromatic amines in cooked meat products. Analytical Methods, 2015. 7(21): 9274–9280.
Resource availability:	NA

## Method details

Heterocyclic amines (HCAs) are one class of harmful compounds that could be produced during meat preparation by grilling. A number of epidemiological studies conducted in rodents have demonstrated the development of cancer in different organs and tissues following dietary exposure to HCAs [2,3]. Most notably, cancers of the colon, liver, oral cavity, hematopoietic system, and lymphoid system [4]. Furthermore, dietary intake of HCAs through red meat consumption has also been shown to increase the risk factor for cancers in humans. Thus, strategies for accurately quantifying HCAs in cooked meats are a major area of research interest to improve the safety of grilled meats.

Analysis of HCAs requires their prior extraction from food matrices. There is a plethora of methods in the literature for extraction of HCAs in cooked foods including liquid–liquid extraction, solid phase extraction (SPE), and tandem solid phase extraction to purify HCAs prior to analysis [[5], [6], [7], [8]]. These methods require efficient tedious sample pre-treatments to remove interfering meat components such as fats, inorganic salts, proteins, and carbohydrates followed by sample pre-concentration steps to detect HCAs which typically occur at low parts-per-billion-levels in cooked foods. This makes existing sample preparation methods for HCA analysis in cooked foods laborious, time-consuming and unsuitable for multiple sample analysis. Pressurized liquid extraction is a promising alternative to traditional HCA extraction methods, where more polar solvents can be used at elevated temperatures and pressures to selectively and efficiently extract target compounds [[9], [10], [11]]. Two analytical methods are at the forefront of HCA analysis in cooked foods including liquid chromatography coupled with mass spectrometry (LC–MS) and gas chromatography-mass spectrometry (GC–MS). GC–MS is sensitive, but prior derivatization of HCAs is necessary for GC analysis, which is time-consuming and not all HCAs can be derivatized completely, limiting the application of GC–MS for HCA analysis. Liquid chromatography-tandem mass spectrometry (LC–MS/MS) is a preferred method, owing to its high selectivity, superior sensitivity and analysis without prior derivatization treatments [12]. To the best of our knowledge, there exist but few methods in the literature that are based on pressurized liquid extraction-liquid chromatography-high resolution accurate mass tandem mass spectrometry (PLE-LC-HRAMS/MS) for HCA analysis [1,5,13]. These methods used dichloromethane/acetonitrile (1:1, v/v) [1] and dichloromethane/acetone, 50/50 (v/v) [13] for HCA extraction followed by filtration of extracted analytes, identification and quantification of HCAs on a liquid chromatography- mass spectrometer. Significantly, these methods were considered to be fast, reliable and require sample pre-treatments using solid phase extraction (SPE), or liquid extraction prior LC/MS analysis.

Our modified pressurized liquid extraction method is based on pure methanol for pressurized solvent extraction of HCAs and UHPLC-HRAMS/MS analysis. In this method, HCA containing samples are digested in basic methanol and selectively extracted on a Dionex ASE 350 extractor (ThemoScientific, Brampton, On, Canada). Extracted analytes are concentrated during extraction, filtered (Mini-UniPrepTM G2 syringeless filter, 0.2 μm, Whatman, Buckinghamshire, UK) and submitted to UHPLC-HRAMS/MS analysis without the need for sample pre-treatments. The method was fast, and showed good accuracy and reliability. Furthermore, use of automated pressurized liquid extractor (Dione ASE 350) would allow for multi-sample analysis of HCAs.

### Reagents and standards

Analytical grade HCA standards were purchased from Toronto Research Chemicals Inc. (Toronto, Canada). These included 2-amino-3-methylimidazo[4,5-f]quinoline (IQ), 2-amino-3,8-dimethylimidazo[4,5-f)quinoxaline (MeIQx), 2-Amino-3,4-dimethylimidazo[4,5-f]quinoline (MeIQ), 2-amino-1-methyl-6-phenylimidazo[4,5-b]pyridine (PhIP), 1-Methyl-9H-pyrido[3,4-b]indole (Harman), 9H-pyrido[3,4-b]indole (Nor-Harman), and 2-amino-3,4,7,8-tetramethylimidazo[4,5-f]quinoxaline (TriMeIQx). Sodium hydroxide was purchased from Sigma Aldrich (Oakville, Ontario, Canada). Dionex ASE™Prep DE diatomaceous earth was obtained from Thermo-Fisher Scientific (Ottawa, Canada).

### Calibration curve for validation of pressurized liquid extraction of heterocyclic amine method

Stock HCAs standard solutions (1 mg/mL) of amine 2-amino-3-methylimidazo[4,5-f]quinoline (IQ), 2-amino-3,8-dimethylimidazo[4,5-f]quinoxaline (MeIQx), 2-Amino-3,4-dimethylimidazo[4,5-f]quinolone (MeIQ), 2-amino-3,4,7,8-tetramethylimidazo[4,5-f]quinoxaline (TriMeIQx), 2-amino-1-methyl-6-phenylimidazo[4,5-b]pyridine (PhIP), 1-Methyl-9-H-pyrido[3,4-b]indole (Harman) and 19H-pyrido[4,3-b]indole (Nor-Harman) were prepared and stored at −80 °C until needed. Six calibration standard solutions (0−100 μg/L) were prepared freshly by serial dilutions of the stock standard solutions using acetonitrile:water (10:90 % v/v) as solvent, with TriMeIQx added as internal standard (final spike concentration = 0.05 μg/mL). The calibration standards (5, 10, 20, 50, 80,100 μg/mL) were prepared in quadruplicates (n = 4).

### Extraction of HCA standards by accelerated solvent extractor (ASE)

Aliquots (**50 μg/L)** of working standard mixture of HCAs containing internal standard (TriMeIQx = 0.05 μg/mL) were used as quality control for the extraction of samples with the accelerated solvent extractor (Dionex ASE 350, Thermo Scientific, MO, USA). Briefly, 25 uL of 5000 μg/L **(5 μg/mL)** working standard mixture was mixed with 2.5 mL of 0.5 M NaOH in MeOH/Water (70:30 v/v) and incubated for 2 h at room temperature with stirring in a 5 mL beaker. Diatomaceous earth (1:2 v/w) was added to the mixture and the paste loaded into 10 mL stainless steel ASE extraction cells for extraction with pure methanol. The ASE extraction program was set as follows: 2 cycles, 5 min heating time, 80 °C extraction temperature, 160 s purge time, and 1606 psi static pressure. The extracted analytes in 20 mL of methanol were evaporated to dryness with a rotary evaporator and nitrogen gas at room temperature. HCAs were re-suspended in 2.5 mL methanol and filtered (Mini-UniPrepTM G2 syringeless filter, 0.2 μm, Whatman, Buckinghamshire, UK) prior to analysis by UHPLC-HRMS/MS. The ASE extractions were performed in quadruplicates (n = 4) for each working standard mixture.

### Ultra-high performance liquid chromatography-high resolution accurate mass tandem spectrometric analysis of HCAs

The UHPLC-HRAMS/MS analysis was performed on a LTQ Orbitrap XL mass spectrometer with an automated Dionex UltiMate 3000 UHPLC system controlled by Chromeleon software (Thermo Scientific, MO, USA). A Luna C18 column (100 × 2.0 mm I.D., particle size: 3 μm, pore diameter: 100 Å) purchased from Phenomenex (CA, USA) was used for HCAs separation. The solvent system used on the analytical column was: H_2_O, 30 mM ammonium formate (pH 3.2) as solvent A and pure acetonitrile as solvent B. Chromatographic separation was carried out at 20 °C with a flow rate of 0.2 mL/min, and 10 μL of sample was injected in the instrument. The gradient used for separation was as follows: 0−1 min 10 % B, 1−3 min 10–20 % B, 3−6 min 20–30 % B, 6−9 min 30–40 % B, 9−12 min 40 % B, 12−13 min 40–50 % B, and re-equilibrated at 90 % A for 2 min. The Orbitrap mass spectrometer was operated in the positive ESI mode. The following optimized parameters were used for the Orbitrap mass spectrometer, sheath gas: 8, auxiliary gas: 2, ion spray voltage: 4.50 kV, capillary temperature: 320 ^0^C; S-lens RF: 100 V; capillary voltage: 30 V, mass range: 100−1000 *m/z*; full scan mode at a resolution of 60,000 *m/z*; top-3 data dependent MS/MS at a resolution of 30,000 *m/z* and collision energy of 35 (arbitrary unit); injection time 15 min; isolation window: 1.5 *m/z*; automatic gain control target: 2 e5 with dynamic exclusion setting of 30.0 s. The mass spectrometer was externally calibrated to 1 ppm using ESI negative and positive calibration solutions (Thermo Scientific, MO, USA) prior to usage.

### Evaluation of accuracy and precision of ASE-UHPLC-HRAMS/MS method for HCA analysis

Working standard mixtures (20, 40 and 60 μg/L) of HCAs containing internal standard (TriMeIQx = 0.05 μg/mL) dissolved in 2.5 mL of 0.5 M NaOH in MeOH/Water (70:30 v/v) and incubated for 2 h at room temperature with stirring. Diatomaceous earth (1:2 v/w) was added to the mixture and the paste loaded into 10 mL stainless steel ASE extraction cells, and extracted with an accelerated solvent extractor (Dionex ASE 350, Thermo Scientific, MO, USA). Extracted analytes were recovered after roto-evaporation, resuspended in 2.5 mL pure methanol and analyzed by UHPLC-HRMS/MS on a LTQ Orbitrap XL mass spectrometer (Thermo Scientific, MO, USA) with an automated Dionex UltiMate 3000 UHPLC system as previously described for HCA standards.

### Method applicability: HCAs in grilled beef and moose meats

Beef (Bovinae) and moose (Cervidae) striploin steaks (longissimus muscle) were obtained from a local market and from Newfoundland and Labrador Department of Natural Resources respectively. Moose steaks were taken from 4 different animals while beef steaks purchased from 4 different batches of beef were used to mitigate any inherent variability of the meat sources. Ethics approval for this study was granted by Memorial University Animal Care Committee as mandated by the Canadian Council on Animal Care, and all the experiments were performed in accordance with relevant guidelines and regulations. Steaks (1 lb) of beef (B) and moose (M) meat from different batches were cut and divided into four replicates (n = 4), and kept under the same conditions until grilling time [14].

### Cooking conditions

Beef and moose samples were grilled at 200−250 °C for 25 min on a grill (Cuisinart® Gourmet 600B, Canadian Tire, NL) reaching an internal temperature of 75 °C. The grill was thoroughly cleaned between samples to avoid any possible cross contamination from meat exudates or trimmings. Meat samples were turned regularly during grilling. After grilling, each replicate was divided into two subsets. One subset was cut into two-inch cubes and used for sensory analysis while the other subset was labeled and stored at −80 °C for HCA analysis [14].

### Extraction of heterocyclic amines in grilled meats

HCAs were extracted with an accelerated solvent extractor (Dionex ASE 350, Thermo Scientific, MO, USA). Grilled meat (1 g) was ground, spiked with internal standard (TriMeIQx = 0.05 μg/mL), mixed with 0.5 M NaOH in MeOH/Water (70:30 v/v) and incubated for 2 h at room temperature. Diatomaceous earth (1:2 w/w) was added to the mixture and HCAs were extracted with pure methanol. The ASE extraction program was as follows: 2 cycles, 5 min heating time, 80 °C extraction temperature, 160 s purge time, and 1606 psi static pressure. The extracted analytes were evaporated to dryness with a rotary evaporator and nitrogen gas at room temperature. HCAs were re-suspended in 2.5 mL methanol and filtered (Mini-UniPrepTM G2 syringeless filter, 0.2 μm, Whatman, Buckinghamshire, UK) prior to ultrahigh performance liquid chromatography coupled to high resolution accurate mass tandem mass spectrometry (UHPLC-HRMS/MS) analysis [14].

### Analysis of heterocyclic amines in grilled meats

The UHPLC-HRMS/MS analysis was performed on a LTQ Orbitrap XL mass spectrometer (Thermo Scientific, MO, USA) as previously described for HCA standards.

## Method validation

**Fig. 1:**
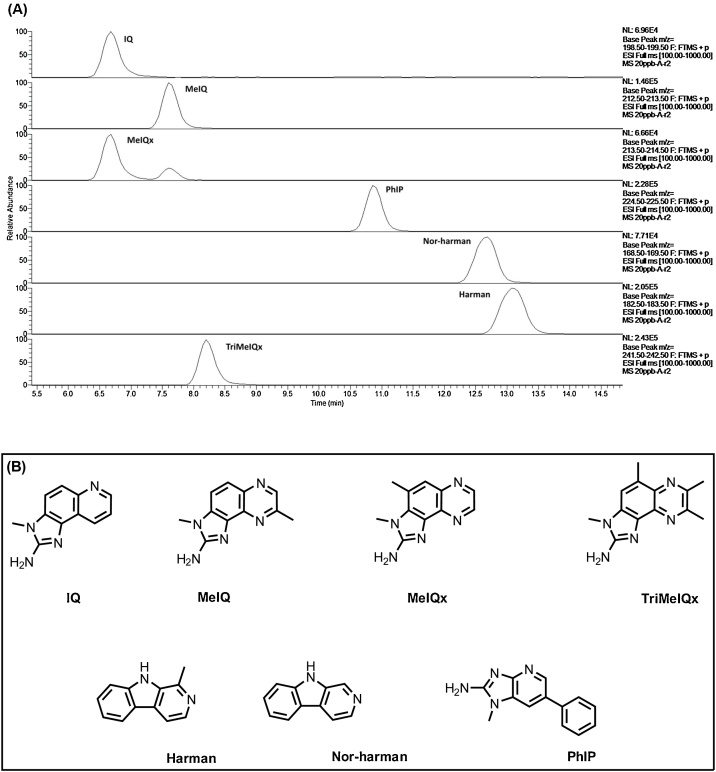
Extracted ion chromatograms (EICs) of HCAs. **(A)** IQ (*m/z* 199.09), MeIQ (*m/z* 213.10), MeIQx (*m/z* 214.10), PhIP (*m/z* 225.10), Nor-harman (*m/z* 169. 07), Harman (*m/z* 183. 08), and TriMeIQx (*m/z* 242.13) standards at 10 μg/L. **(B)** Chemical structures of seven (7) HCAs. IQ = 2-amino-3-methylimidazo[4,5-f]quinolone. MeIQx = 2-amino-3,8-dimethylimidazo[4,5-f]quinoxaline; MeIQ = 2-Amino-3,4-dimethylimidazo[4,5-f]quinolone; PhIP = 2-amino-1-methyl-6 phenylimidazo[4,5-b]pyridine; Harman = 1-Methyl-9H-pyrido[3,4-b]indole; Nor-Harman = 9H-pyrido[3,4-b]indole; TriMeIQx = 2-amino-3,4,7,8-tetramethylimidazo[4,5-f]quinoxaline. HCA = Heterocyclic amine.

Ultra-high performance liquid chromatograph-high resolution accurate mass spectrometry (UHPLC-HRAMS) with selected ion monitoring (SIM) acquisition was performed for method validation including linear regression, limit of detection (LOD), limit of quantitation (LOQ), precision, accuracy and recovery. Fig. 1A-B shows the extracted ion chromatograms and chemical structures of HCA standards evaluated, and demonstrate the suitability of UHPLC-HRMS/MS to resolve and accurately detect all 7 HCA compounds using a Luna C18 column. The high resolution mass spectrometric capability of the LTQ Orbitrap XL mass spectrometer allowed for accurate and unambiguous identification and quantitation of individual HCA resolved in the chromatograms (Table 1). All HCAs evaluated in our method were identified based on their mass spectra and fragment ions derived from MS/MS. The two co-eluted peaks at 6.68 min. corresponding to IQ and MeIQx were resolved and quantify by extracted ion chromatography (XIC) based on the exact masses of precursor ions; [M+H] ^+^, which for IQ and MeIQx were different: *m/z* (IQ) = 199.0978; *m/z* (MeIQx) = 214.1083. For quantitation, the area under each peak corresponding to each precursor ion exact mass was measured using Xcalibur version 4.0 (Thermo Scientific, MO, USA). Thus, both masses of precursor ion and retention/elution times were used for peak identification and quantitation of IQ and MeIQx.

**Table 1:** Exact masses of HCAs with elemental compositions as obtained following analysis by Ultra high performance liquid chromatography coupled to high resolution accurate mass tandem mass spectrometry (UHPLC-HRAMS).

HCA	Assignment	Ion	MS^n^	Elemental Composition	Fragment Ion (m/z)	Measured Ion (m/z)	Calculated (m/z)	Error (mDA)	Error (ppm)
TriMeIQx	[M+H]^+^	A	MS^1^	C_13_H_15_N_5_	242^a^	242.1377 (100)	242.1373	0.4	1.5224
	A-15	B	MS^2^	C_12_H_13_N_5_	227^b^	227.1143 (100)	227.1139	0.5	2.0574
IQ	[M+H]^+^	A	MS^1^	C_11_H_10_N_4_	199^a^	199.0959 (100)	199.0965	−0.6	−2.9331
	A-15	B	MS^2^	C_10_H_7_N_4_	184^b^	184.0725 (95)	184.0730	−0.5	−2.8024
MeIQx	[M+H]^+^	A	MS^1^	C_11_H_11_N_5_	214^a^	214.1067 (100)	214.1060	0.6	2.8823
	B-41	B	MS^2^	C_9_H_8_N_4_	199^b^	173.0804 (91)	173.0808	−0.5	−2.6292
MeIQ	[M+H]^+^	A	MS^1^	C_12_H_12_N_4_	213^a^	213.1114 (100)	213.1108	0.6	2.8897
	A-15	B	MS^2^	C_11_H_9_N_4_	198^b^	198.0881 (100)	198.0887	−0.5	−2.7307
PhIP	[M+H]^+^	A	MS^1^	C_13_H_12_N_4_	225^a^	225.1113 (100)	225.1108	0.5	−2.9766
	A-15	B	MS^2^	C_12_H_9_N_4_	210^b^	210.0880 (20)	210.0887	−0.6	−3.0831
Harman	[M+H]^+^	A	MS^1^	C_12_H_10_N_2_	183^a^	183.0899 (100)	183.0890	0.9	5.0535
	A-15	B	MS^2^	C_10_H_7_N_4_	168^b^	168.0186 (51)	168.0192	−0.6	−3.7960
Nor-Harman	[M+H]^+^	A	MS^1^	C_11_H_8_N_2_	169^a^	169.0744 (48)	169.0733	1.1	6.4610
	A-54	B	MS^2^	NA	115^b^	115.1132 (3 %)	115.0530	−1.2	−10.697

HCA = Heterocyclic amine. IQ = 2-amino-3-methylimidazo[4,5-f]quinolone. MeIQx = 2-amino-3,8-dimethylimidazo[4,5-f]quinoxaline; MeIQ = 2-Amino-3,4-dimethylimidazo[4,5-f]quinolone; PhIP = 2-amino-1-methyl-6 phenylimidazo[4,5-b]pyridine; Harman = 1-Methyl-9H-pyrido[3,4-b]indole; Nor-Harman = 9H-pyrido[3,4-b]indole; TriMeIQx = 2-amino-3,4,7,8-tetramethylimidazo[4,5-f]quinoxaline. ESI-positive mode. Data obtained on a Dionex UHPLC 3000 system coupled a ThermoScientific LTQ Orbitrap XL™ hybrid Fourier Transform mass spectrometer (FTMS). n = 4 per experimental replicate.

a Denotes precursor ion; [M+H] ^+^.

b Denotes product ion.

Significantly, complete resolution/discrimination of co-eluates (IQ and MeIQx) based on their different exact masses was made possible due in part to the high resolving power of the LTQ Orbitrap XL mass spectrometer used in acquiring mass data in our accelerated solvent extraction-ultra high pressure-high resolution accurate mass/mas spectrometric (ASE-UHPLC-HRAMS/MS) method. The LTQ Orbitrap XL mass spectrometer (Thermo Scientific, MO, USA) used in our method has a resolving power (R) up to *m/z* = 100,000. Mass data for ASE-UHPLC-HRAMS/MS method for HCA analysis described was acquired on LTQ Orbitrap XL mass spectrometer (Thermo Scientific, MO, USA) operating in positive mode at resolving power (R) *m/z* = 60,000. Similar applications of accurate mass high resolution mass spectrometry to resolve co-eluted chromatographic peaks based on masses of co-eluates have been reported [15]. The exact mass of IQ and MeIQx calculated based on chemical structure and chemical formula using ChemDraw (PerkinElmer, Version 16.0.1.4, Massachusetts, U.S.A.) was 198.09055 and 213.10145 respectively.

**Fig. 2:**
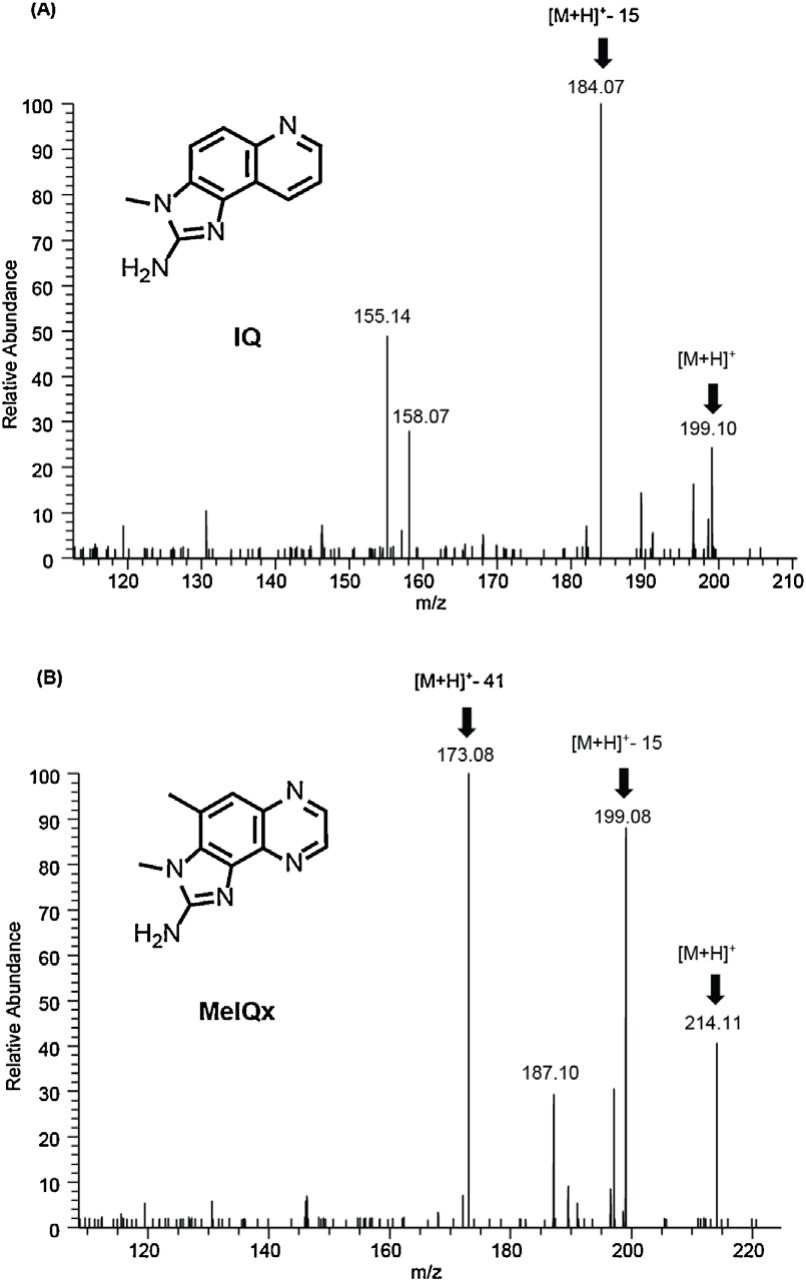
HRAMS/MS spectra of co-eluted HCAs showing diagnostic fragment ions. **(A)** Tandem MS/MS mass spectra of IQ **(B)** Tandem MS/MS mass spectra of MeIQx. IQ = 2-amino-3-methylimidazo[4,5-f]quinolone. MeIQx = 2-amino-3,8-dimethylimidazo[4,5-f]quinoxaline. M = Exact mass of fragment ion. HCA = Heterocyclic amine.

Furthermore, identification of IQ and MeIQx was corroborated based on the MS/MS spectra which showed distinctly different fragmentation patterns and diagnostic fragments ions which matched the literature MS values for IQ and MeIQx (see Fig. 2A-B). For IQ, the diagnostic product ion at *m/z* = 184.0743 formed by loss of methyl unit (−CH_3_) from precursor/parent ion at *m/z* = 199.0978 [1,5,16]. For MeIQx, the product ions at *m/z* = 199.0852 resulted from loss of one methyl unit (−CH_3_) from the precursor/parent ion, and *m/z* = 173.0822 corresponded to elimination of one amino imidazole moiety (–C_2_NH_3_) from the precursor/parent ion at *m/z* 214.1083 [1,5,16]. These diagnostic fragment ions were in good agreement with the reported literature values used to identify and quantify IQ and MeIQx [1,5,16].

**Fig. 3:**
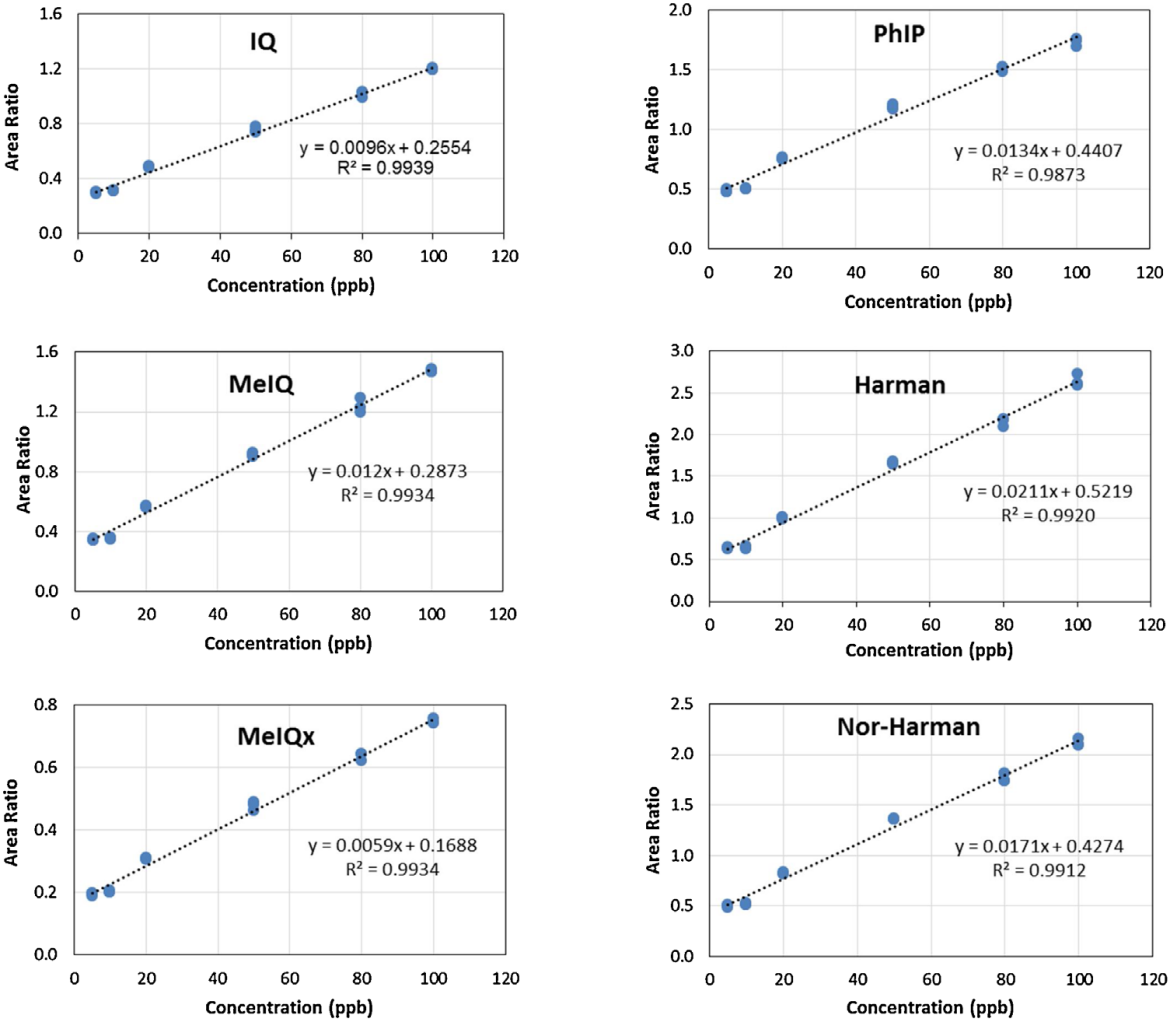
Calibration curves for 6 heterocyclic amine standards. IQ = 2-amino-3-methylimidazo[4,5-f]quinolone. MeIQx = 2-amino-3,8-dimethylimidazo[4,5-f]quinoxaline; MeIQ = 2-Amino-3,4-dimethylimidazo[4,5-f]quinolone; PhIP = 2-amino-1-methyl-6 phenylimidazo[4,5-b]pyridine; Harman = 1-Methyl-9H-pyrido[3,4-b]indole; Nor-Harman = 9H-pyrido[3,4-b]indole; TriMeIQx = 2-amino-3,4,7,8-tetramethylimidazo[4,5-f]quinoxaline.

Calibration curves for the 7 HCA standards showed good linearity over the range 20–100 μg/L (Table 2 and Fig. 3). The linearity gives an indication of the appropriateness of a method, and ideally this relationship should be perfectly linear. In line with this, our method showed good linearity for all 7 HCA compounds with linear correlation coefficients greater than 0.991 which means there is good correlation between peak areas and concentrations (Table 2 and Fig. 3). Limits of detection (LOD) and quantification (LOQ) were determined using: LOD 3(SD of intercept/Slope) and LOQ 10(SD of intercept/Slope) [17]. LODs ranged from 8.2 to 12 μg/L HCAs, setting a lower limit on HCA concentration in solution and were accompanied by recoveries ranging from 63.5 to 100% suggesting that the ASE extraction method for HCA compounds in accurate and efficient (Table 2).

**Table 2:** Quality control test of six HCAs observed in grilled moose and beef steaks.

HCAs	Rt (min)	Linear Regression Equation	Linear range (μg/L)	Correlation Coefficient (R²)	LOD (μg/L)	LOQ (μg/L)	Recovery %
IQ	6.68	Y = 0.255382 + 0.00955059*X	20–100	0.9939	8.2180	27.3933	79.5
MeIQx	7.60	Y = 0.168785 + 0.00587124*X	20–100	0.9934	8.5163	28.3877	88.5
MeIQ	6.68	Y = 0.287259 + 0.0119956*X	20–100	0.9934	8.4972	28.3240	*quant.*
PhIP	10.86	Y = 0.440687 + 0.0133516*X	20–100	0.9873	11.8526	39.5088	98.9
Harman	13.12	Y = 0.52188 + 0.0211374*X	20–100	0.9920	9.4144	31.3812	79.9
Nor-Harman	12.74	Y = 0.427437 + 0.0170845*X	20–100	0.9912	9.8680	32.8935	63.5

HCA = Heterocyclic amine. IQ = 2-amino-3-methylimidazo[4,5-f]quinolone. MeIQx = 2-amino-3,8-dimethylimidazo[4,5-f]quinoxaline; MeIQ = 2-Amino-3,4-dimethylimidazo[4,5-f]quinolone; PhIP = 2-amino-1-methyl-6 phenylimidazo[4,5-b]pyridine; Harman = 1-Methyl-9H-pyrido[3,4-b]indole; Nor-Harman = 9H-pyrido[3,4-b]indole; TriMeIQx = 2-amino-3,4,7,8-tetramethylimidazo[4,5-f]quinoxaline. LOD = Limit of detection [17]; LOQ = Limit of quantitation [17]. N = Number of calibration standards (5, 10, 20, 50, 80, 100 μg/L). Spike level = 50 μg/L. N = 6 calibration standards.

$\text{S}\text{D}\;\text{o}\text{f}\;\text{I}\text{n}\text{t}\text{e}\text{r}\text{c}\text{e}\text{p}\text{t}=\;\text{S}\text{E}\;\text{o}\text{f}\;\text{I}\text{n}\text{t}\text{e}\text{r}\text{c}\text{e}\text{p}\text{t}\text{*}\sqrt{\text{N}}$

(where N = 6)

$\text{L}\text{O}\text{D}=\;3\;\frac{\text{S}\text{D}\;\text{o}\text{f}\;\text{I}\text{n}\text{t}\text{e}\text{r}\text{c}\text{e}\text{p}\text{t}}{\text{S}\text{l}\text{o}\text{p}\text{e}}$

$\text{L}\text{O}\text{Q}=\;10\;\frac{\text{S}\text{D}\;\text{o}\text{f}\;\text{I}\text{n}\text{t}\text{e}\text{r}\text{c}\text{e}\text{p}\text{t}}{\text{S}\text{l}\text{o}\text{p}\text{e}}$

$\text{P}\text{e}\text{r}\text{c}\text{e}\text{n}\text{t}\;\text{r}\text{e}\text{c}\text{o}\text{v}\text{e}\text{r}\text{y}\;\left(\text{\%}\right)=\;100\text{*}\;\frac{\text{R}\text{e}\text{c}\text{o}\text{v}\text{e}\text{r}\text{e}\text{d}\;\text{l}\text{e}\text{v}\text{e}\text{l}}{\text{S}\text{p}\text{i}\text{k}\text{e}\;\text{l}\text{e}\text{v}\text{e}\text{l}}$

**Fig. 4:**
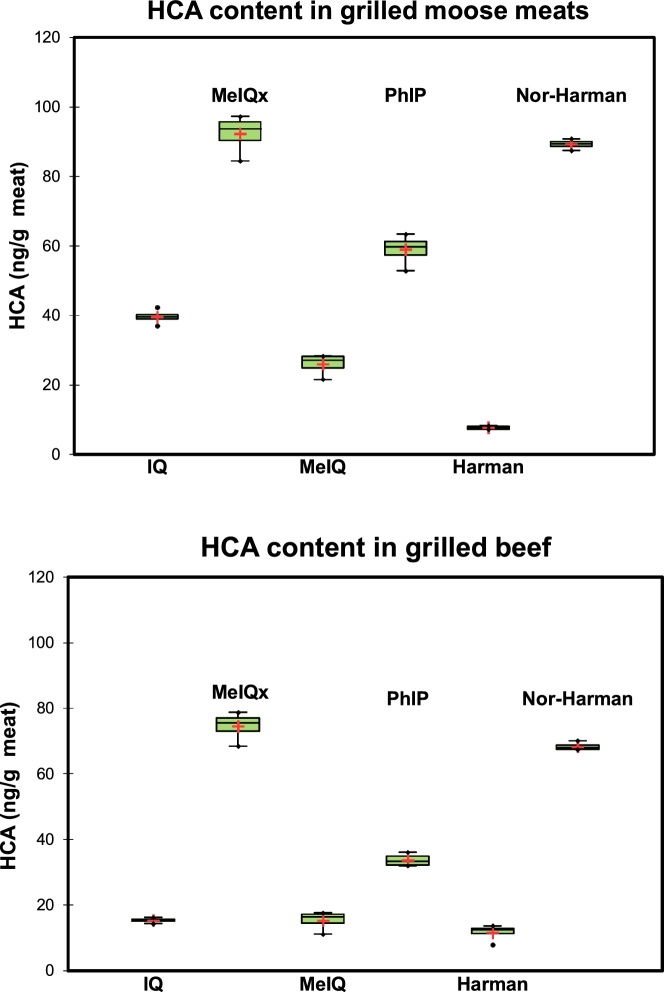
Box plot showing heterocyclic amine (HCA) level (ng/g meat) in grilled moose or beef. IQ = 2-amino-3-methylimidazo[4,5-f]quinoline, MeIQx = 2-amino-3,8- dimethylimidazo[4,5-f)quinoxaline, MeIQ = 2-Amino-3,4-dimethylimidazo[4,5-f]quinolone, PhIP = 2-amino-1-methyl-6-phenylimidazo[4,5-*b*]pyridine. Replication (n) = 4. HCA = Heterocyclic amine.

Application of UHPLC-HRAMS to grilled beef and moose meats showed results consistent with that reported in the literature (Fig. 4). The most abundant HCA detected was MeIQx consistent with findings in other cooked meat studies [18]. The formation of HCAs in thermally treated meats is based on many factors including cooking methods, temperature, duration of cooking, and type of meat [19].

The intra-day precision and accuracy of the method were evaluated at 3 quality control levels (40, 60 and 100 μg/L) which were above the LOD, LOQ and within linear ranges for HCAs (Table 3). The intra-day precision was less good and showed accuracies from 45.4 to 97.1%. The detailed values of intra-day precision and accuracy are shown in Table 3. All the values are within an acceptable range.

**Table 3:** Intra day precision and accuracy of 6 HCAs following extraction by accelerated solvent extractors and confirmation of composition and levels by ultra high performance liquid chromatography coupled to high resolution accurate mass tandem mass spectrometry (UHPLC-HRAMS).

HCA	Spike level (μg/L)	Mean Recovered level (μg/L)	SD	SE	Recovery (%)
IQ	80	61.91	2.28	0.93	77.39
	60	44.16	7.30	2.98	73.59
	40	35.32	2.88	1.17	88.29
MeIQx	80	35.85	2.66	1.08	48.57
	60	27.22	2.02	0.83	45.37
	40	22.90	2.18	0.89	57.25
MeIQ	80	65.67	4.18	1.71	82.08
	60	47.24	4.65	1.90	78.74
	40	38.17	1.53	0.63	94.42
PhIP	80	70.62	4.44	1.81	88.28
	60	52.48	4.35	1.78	87.46
	40	40.31	8.07	3.29	100.77
Harman	80	73.33	13.02	5.32	91.67
	60	58.28	8.69	3.55	97.14
	40	32.94	13.32	5.44	82.35
Nor-harman	80	62.45	7.81	3.19	78.06
	60	48.49	3.43	1.40	80.82
	40	29.41	10.38	4.24	73.53

HCA = Heterocyclic amine. IQ = 2-amino-3-methylimidazo[4,5-f]quinolone. MeIQx = 2-amino-3,8-dimethylimidazo[4,5-f]quinoxaline; MeIQ = 2-Amino-3,4-dimethylimidazo[4,5-f]quinolone; PhIP = 2-amino-1-methyl-6 phenylimidazo[4,5-b]pyridine; Harman = 1-Methyl-9H-pyrido[3,4-b]indole; Nor-Harman = 9H-pyrido[3,4-b]indole; TriMeIQx = 2-amino-3,4,7,8-tetramethylimidazo[4,5-f]quinoxaline. SD = Standard deviation; SE = Standard error; RSD = Relative standard deviation. Replication (n) = 6. Data obtained on a Dionex UHPLC 3000 system coupled a ThermoScientific LTQ Orbitrap XL™ hybrid Fourier Transform mass spectrometer (FTMS). Extraction done on ThermoScientific Dionex 350 Accelerated Solvent Extractor.

Matrix effects in grilled moose samples caused ion signal suppression for IQ, MeIQ, and Harman whereas the ion signals for MeIQx, PhIP and Nor-harman were enhanced (Table 4). In grilled beef samples, the matrix had suppressing effect on ion signals for IQ, MeIQ, PhIP and Harman whereas the ion signals for MeIQx and Nor-harman were supressed which reduced the signal strengths (Table 5). Similar signal suppression and enhancement effects has been reported for LC–MS analysis of HCAs in meats including pork [16]. In the ASE-UHPLC-HRAMS/MS method described, HCA standard solutions and samples were spiked with TriMeIQx, as the internal standard for quantification, and normalized peaks areas of standards and samples based on internal standard (i.e. ratios) used to calculate concentrations of standard solutions and samples to minimize possible quantification errors produced by the matrix effects.

**Table 4:** Evaluation of meat matrix effects for HCA analysis by Ultra high performance liquid chromatography coupled to high resolution accurate mass tandem mass spectrometry (UHPLC-HRAMS) in grilled moose.

Moose	IQ	MeIQ	MeIQx	PhIP	Harman	Nor-Harman
MU-r1	93.31	54.31	181.65	131.76	21.58	187.98
MU-r2	73.98	43.08	193.97	68.76	16.51	178.21
MU-r3	83.65	59.58	200.18	131.16	16.69	187.12
MU-r4	78.81	60.27	200.06	110.56	18.26	188.68
%ME	82.44	54.31	193.97	110.56	18.26	185.49

IQ = 2-amino-3-methylimidazo[4,5-f]quinolone. MeIQx = 2-amino-3,8-dimethylimidazo[4,5-f]quinoxaline; MeIQ = 2-Amino-3,4-dimethylimidazo[4,5-f]quinolone; PhIP = 2-amino-1-methyl-6 phenylimidazo[4,5-b]pyridine; Harman = 1-Methyl-9H-pyrido[3,4-b]indole; Nor-Harman = 9H-pyrido[3,4-b]indole; TriMeIQx = 2-amino-3,4,7,8-tetramethylimidazo[4,5-f]quinoxaline. ME = Matrix effect

The grilled meat matrix effect was determined using the post-extraction method based on the formula below [20]:

$\text{M}\text{E}=\;\frac{\text{B}\;}{\text{A}}\;\text{*}\;100$

Where,

A is the normalized peak area/concentration of an analyte in a standard solution. B is the normalized peak area/concentration of the analyte in grilled meat samples. All standard solutions and grilled meat samples where spiked with internal standard and their corresponding peak areas normalized based on the area ratios.

If,

ME ∼ 100 %, there is no matrix effect. If ME < 100 %, an ion-suppression occurred and, if ME > 100 %, ion-enhancement occurred.

**Table 5:** Evaluation of meat matrix effects for HCA analysis by Ultra high performance liquid chromatography coupled to high resolution accurate mass tandem mass spectrometry (UHPLC-HRAMS) in grilled beef.

Beef	IQ	MeIQ	MeIQx	PhIP	Harman	Nor-Harman
BU-r1	42.21	45.21	248.09	76.55	24.49	154.14
BU-r2	30.16	23.71	139.59	65.87	26.31	137.62
BU-r3	58.95	31.21	153.19	72.41	25.36	135.23
BU-r4	32.61	36.03	186.76	71.27	29.07	143.51
%ME	40.99	34.04	181.91	71.52	26.31	142.62

IQ = 2-amino-3-methylimidazo[4,5-f]quinolone. MeIQx = 2-amino-3,8-dimethylimidazo[4,5-f]quinoxaline; MeIQ = 2-Amino-3,4-dimethylimidazo[4,5-f]quinolone; PhIP = 2-amino-1-methyl-6 phenylimidazo[4,5-b]pyridine; Harman = 1-Methyl-9H-pyrido[3,4-b]indole; Nor-Harman = 9H-pyrido[3,4-b]indole; TriMeIQx = 2-amino-3,4,7,8-tetramethylimidazo[4,5-f]quinoxaline. ME = Matrix effect.

## Declaration of Competing Interest

The authors declare no conflict of interest.
